# Artificial intelligence-based automated determination in breast and colon cancer and distinction between atypical and typical mitosis using a cloud-based platform

**DOI:** 10.3389/pore.2024.1611815

**Published:** 2024-10-30

**Authors:** Nilay Bakoglu, Emine Cesmecioglu, Hirotsugu Sakamoto, Masao Yoshida, Takashi Ohnishi, Seung-Yi Lee, Lindsey Smith, Yukako Yagi

**Affiliations:** ^1^ Department of Pathology, Laboratory Medicine, Memorial Sloan Kettering Cancer Center, New York, NY, United States; ^2^ Department of Pathology, Marmara University Research and Education Hospital, Istanbul, Türkiye; ^3^ Department of Medicine, Division of Gastroenterology, Jichi Medical University, Tochigi, Japan; ^4^ Division of Endoscopy, Shizuoka Cancer Center, Shizuoka, Japan; ^5^ Aiforia Technologies, Cambridge, MA, United States; ^6^ Flywheel.io, Minneapolis, MN, United States

**Keywords:** mitosis, colon adenecarcinoma, breast, artificial intelligence, invasive carcinoma

## Abstract

Artificial intelligence (AI) technology in pathology has been utilized in many areas and requires supervised machine learning. Notably, the annotations that define the ground truth for the identification of different confusing process pathologies, vary from study to study. In this study, we present our findings in the detection of invasive breast cancer for the IHC/ISH assessment system, along with the automated analysis of each tissue layer, cancer type, etc. in colorectal specimens. Additionally, models for the detection of atypical and typical mitosis in several organs were developed using existing whole-slide image (WSI) sets from other AI projects. All H&E slides were scanned by different scanners with a resolution of 0.12–0.50 μm/pixel, and then uploaded to a cloud-based AI platform. Convolutional neural networks (CNN) training sets consisted of invasive carcinoma, atypical and typical mitosis, and colonic tissue elements (mucosa-epithelium, lamina propria, muscularis mucosa, submucosa, muscularis propria, subserosa, vessels, and lymph nodes). In total, 59 WSIs from 59 breast cases, 217 WSIs from 54 colon cases, and 28 WSIs from 23 different types of tumor cases with relatively higher amounts of mitosis were annotated for the training. The harmonic average of precision and sensitivity was scored as F1 by AI. The final AI models of the Breast Project showed an F1 score of 94.49% for Invasive carcinoma. The mitosis project showed F1 scores of 80.18%, 97.40%, and 97.68% for mitosis, atypical, and typical mitosis layers, respectively. Overall F1 scores for the current results of the colon project were 90.02% for invasive carcinoma, 94.81% for the submucosa layer, and 98.02% for vessels and lymph nodes. After the training and optimization of the AI models and validation of each model, external validators evaluated the results of the AI models via blind-reader tasks. The AI models developed in this study were able to identify tumor foci, distinguish *in situ* areas, define colonic layers, detect vessels and lymph nodes, and catch the difference between atypical and typical mitosis. All results were exported for integration into our in-house applications for breast cancer and AI model development for both whole-block and whole-slide image-based 3D imaging assessment.

## Introduction

In recent years, machine learning-based image analysis methodologies have been actively developed in the field of digital pathology to aid the quantification of diagnostic parameters in histology and tumor morphology [1]. Specifically, deep learning is a machine learning methodology that has an accelerated ability to assist in pathological diagnosis [2]. It can provide objective and common metrics for uncertain diagnoses, and more importantly, it has the potential to add new information that could not have been quantified previously by pathologists. Recent studies support that the majority of histopathological malignancies can be diagnosed with high accuracy with deep learning methods [3].

Artificial intelligence (AI) in pathology has important implications not only for diagnostics, but also for cancer research, clinical trials and AI-enabled therapeutic targeting [4]. The involvement of AI in a number of diagnostic tasks using WSIs has expanded rapidly in the last year [5–7]. Achieving the identification of cancer is remarkable. In a study conducted by Bejnordi and colleagues in 2017, out of 32 AI models developed for breast cancer detection in lymph nodes, the best model achieved a human-like area under the curve (AUC) of 0.994 (95% CI 0.983–0.999) [8]. In 2020, Thakur et al reviewed 30 studies related to colorectal cancer, some of which showed high diagnostic accuracy, but the overall study size was limited [9].

AI and other neural networks have made great progress in capturing the detection of morphological diagnoses by pathologists with digital platforms [10]. Diagnostic protocols focus equally on efficiency and accuracy [11]. Recently, code-free and user-friendly solutions have become commercially available to assist in the design, training, validation, and deployment of AI models for pathologists without prior knowledge of machine learning and image processing. Our research group has utilized these code-free solutions to develop in-house AI models using Aiforia’s cloud-based platform. We also investigated their availability, convenience, performance, and expansivity in general digital pathology applications. We chose Aiforia because it is easy to use, accessible, fast, and integrated for our research studies. In this paper, we report three AI models that aided the analysis of prognostic parameters detection of Breast and Colon cancer specimens, and mitosis detection in several organs.

Breast cancer is the most common cancer in women [12]. One of the factors determining the treatment of breast carcinomas is the results of molecular tests performed in invasive carcinoma areas [13]. The assessment of HER2 gene amplification, a key molecular test, is made under a microscope using the *in situ* hybridization (ISH) method in invasive tumor areas [14]. Separating the invasive tumor area from the non-invasive area in the breast tissue is important because it determines the diagnosis and grade of the tumor, and most importantly, it determines the targeted therapy based on the HER-2 status in the tumor cells [15]. Digital pathology provides a cutting-edge solution where algorithms can recognize and select invasive tumor areas. Even more powerful is the ability to combine the above pan-tumor detection with immunohistochemistry (IHC) and ISH studies, considering the studies on semi-automated HER2 gene amplification diagnosis made in invasive tumor areas [16].

Colorectal carcinoma is the third most common cancer and the second most deadly cancer in the world [12]. Endoscopic submucosal dissection (ESD) is a method that makes an early diagnosis and treatment of colorectal and gastric neoplasms [17]. ESD maintains its superiority for lesions with superficial submucosal invasion [18]. The depth of the invasion less than 1,000 µm below the muscularis mucosa is suggested as a cure by The Japan Gastroenterological Endoscopy Society [19]. The factors that determine the pathological stage classification of colorectal cancer are tumor extension in the colonic layers, lymph node metastasis and tumor deposits, distant metastasis, tumor budding, lymph vascular invasion, and perineural invasion, in addition to histological type and grade. With the help of AI model-assisted analysis, we aimed to identify prognostic quantitative parameters on colon resection specimens and integrate the algorithm into our 3D tools (Micro-CT) to improve the quality of virtual H&E.

Mitotic count in histological preparations is associated with tumor growth and grading, has prognostic importance [20], and remains a fundamental part of pathology reports from diagnosis to treatment in all cancers [21]. Currently, the number of mitotic cells is determined by manual counting under the microscope, which is encumbered by inter- and intra-observer variability [22]. This variability causes not only the rate of variability among the mitotic counts by pathologists to differ but also contributes to methodological diversity [23, 24]. Atypical mitotic figures are frequently seen in proliferative and aggressive tumors and, in practice, play an important role in the distinction between benign and malignant tumors [25]. To avoid subjective and laborious counting of mitoses, and to predict prognosis, we developed an AI model to detect mitoses and to distinguish between atypical and typical mitoses.

In this study, we describe how we have been developing AI models using Aiforia’s cloud-based AI solutions for breast and colorectal cancer diagnosis, mitosis detection, and in-house AI application development for our integration of AI-based applications and 3D imaging systems.

## Materials and methods

### Dataset

The studies involving human participants were reviewed and approved by the Institutional Review Board at Memorial Sloan Kettering 18-013. The patients provided written informed consent to participate in this study. Cases were diagnosed and reported by pathologists at the Department of Pathology at Memorial Sloan Kettering Cancer Center. In total, 65 digital slides of 41 ESD specimens diagnosed as low-grade dysplasia (LGD), high-grade dysplasia (HGD), and adenocarcinoma, 102 digital slides of 13 colon resection specimens diagnosed as Mucinous adenocarcinoma for the Colon Project, 141 digital slides of 141 breast specimens diagnosed as Breast Invasive carcinoma, 10 digital slides of 10 breast specimens diagnosed as Benign breast parenchyma for the Breast Project, 40 digital slides of 23 multi-organ tumor specimens (four spleen Neuroendocrine tumors (NETs), four pancreatic NETs, five soft tissue-bone tumors, and ten breast tumors) for the Mitosis Project were used in our study cohort. In total 358 digital slides were trained (348 digital slides were used- some of the same slides were trained in different projects), 158 digital slides were tested, and 49 digital slides were validated across all projects. The same slides were used for training in different projects in the colon resection project (Supplementary Table S1) and some of the slides tested in the colon ESD and Colon resection Projects were also used for validation (Table 1).

**TABLE 1:** Dataset of each project.

Projects	Cases	Diagnosis	Scanner	Total WSI	Trained WSI	Tested WSI	Validated WSI
Mitosis	23	Multiple tumors^a^	P1000	40	28	8	4 (20)^b^
Breast-Invasive	141	Invasive carcinoma	P250 P1000 GT450	141	59	73	9 (19)
Breast Benign	10	Benign breast tissue	GT450	10	—	10	—
Colon-ESD	41	LGD, HGD, Adenocarcinoma	S60	65	53	12	5 (30)
Colon-Resection	13	Mucinous adenocarcinoma	P1000 S60	102	200^c^	55	15 (19 × 10) (CRP-1) 16 (20 × 2) (CRP-2)

WSI, whole slide image; ESD, endoscopic submucosal dissection; LGD, Low-Grade Dysplasia; HGD, High-Grade Dysplasia; CRP, Colorectal project.

^a^ Four splenic Neuroendocrine Tumors (NETs), four pancreatic NETs, five soft tissue-bone tumors, and ten breast tumors.

^b^ The number in the parentheses represents the number of ROIs validated in the WSIs (20 ROIs in 4 images were validated and scored by external validators in the mitosis project).

^c^ Colon Resection specimens were prepared as Whole Mount Slides, scanned by S60 and p1000 scanners, and trained, tested, and validated as WMIs. Some of the same slides were trained for different layers in the project.

### Sample digitization

All the slides were prepared from archival formalin-fixed paraffin-embedded (FFPE) tissue blocks sectioned at 4 µm and stained with Hematoxylin and Eosin (H&E). All H&E slides for all three projects were digitized by four different scanners by depending on availability. The first scanner was a Panoramic P250 (3DHistech Ltd., Budapest, Hungary), which was used with a ×20 objective (NA 0.8) and a pixel resolution of 0.17 μm. The second scanner was a Panoramic P1000 (3DHistech Ltd., Budapest, Hungary), which was used with a ×40 objective (NA 0.95) and a pixel resolution of 0.13 μm. The third scanner was the Aperio GT 450 (Leica Biosystems, Illinois, United States), which was used at ×40 equivalent magnification with a pixel resolution of 0.26 μm. The fourth scanner was a NanoZoomer S60 (Hamamatsu Photonics K.K., Shizuoka, Japan) used at ×40 equivalent magnification with a pixel resolution of 0.23 µm. Especially, in the case of colorectal samples, whole mount slides, whose size is twice the size of regular slides, were used and scanned by NanoZoomer S60 and Panoramic P1000.

### Development of the AI model

**FIGURE 1:**
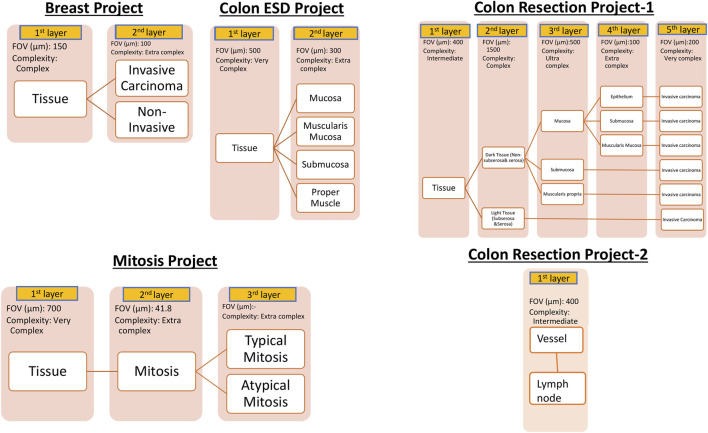
Hierarchical layered structure of AI models. Yellow colors represent each child layer. White colors correspond to the analytical results shown in the results session. User-adjustable parameters on the Aiforia platform are listed below the layer boxes. Augmentation was utilized to further improve the AI model as needed.

**FIGURE 2:**
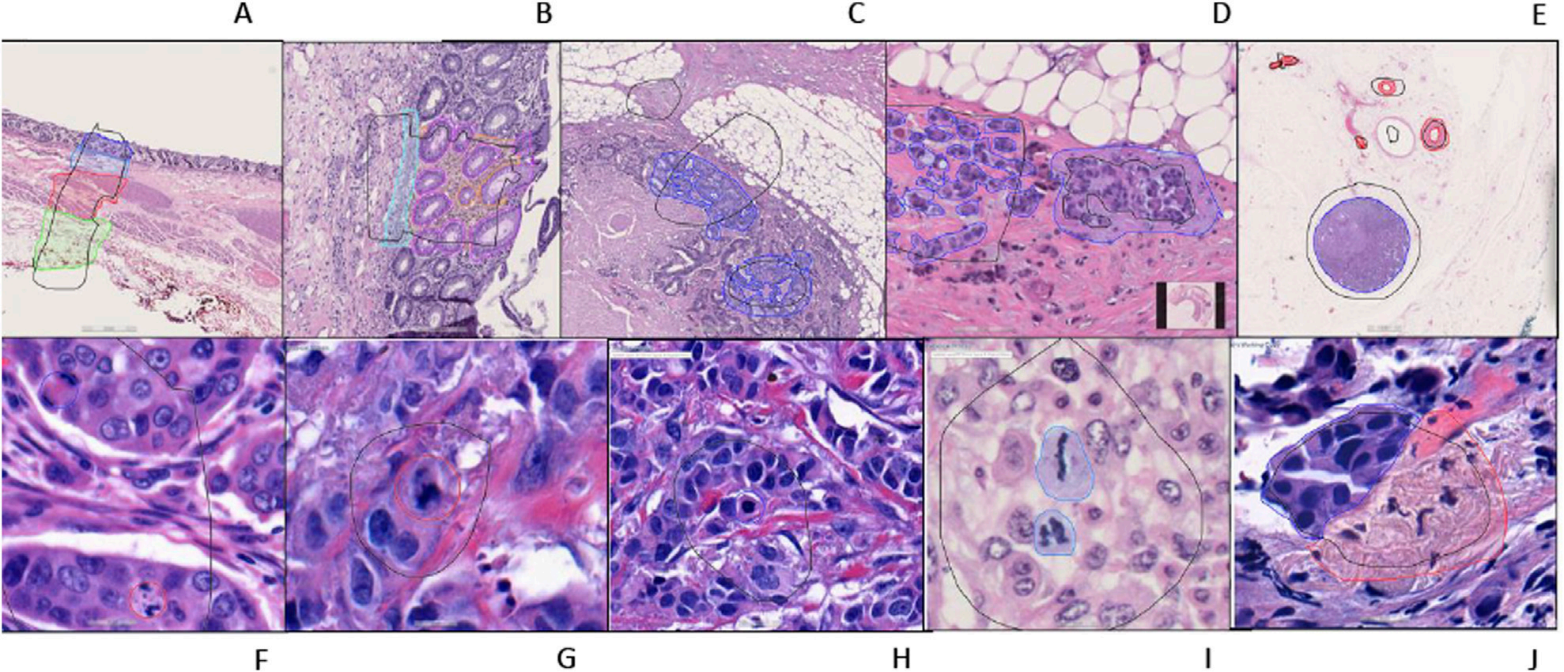
An example of training regions and training annotations **(A–I)**. First row from left to right: **(A–E)**, second row from left to right: **(F–J)**. **(A):** Location type annotation of colonic layers, **(B)**: Colon mucosa layers annotations, **(C, D)**: Colon invasive carcinoma annotations **(E)**: Whole shape lymph node and vessel annotations **(F–H)**: Atypical and typical mitosis annotations (Red: atypical; Blue typical) **(I)**: Mitosis annotation **(J)**: Breast invasive carcinoma annotation [**(A–E, J)** are semantic segmentation annotations, **(F–H)** are object annotations].

For AI model development, Aiforia’s cloud-based platform (Aiforia Technologies, Cambridge, MA, United States) was used. The AI-model training dataset representing the entire dataset was carefully selected. These whole slide images were uploaded to Aiforia’s cloud platform in their native file formats (mrxs, svs, or ndpi) and automatically converted into platform-compatible file types. Using the training dataset, AI projects were created, and the AI models were custom-designed and presented as a graphic layer tree. Each layer consists of an individual convolutional neural network (CNN) that performs a task (segmentation or object detection) with adjustable parameters, such as field of view, and resolution of the input patch image. The structure of the AI models is shown in Figure 1. The Breast Carcinoma AI model was built with three classes: Tissue, Invasive tumor, and non-invasive tumor. The invasive tumor area included IHC/ISH target cells, and the Non-invasive tumor area consisted of tumor stroma, necrosis, non-neoplastic breast, and non-invasive lesions. For the Colon ESD project, mucosa, muscularis mucosa, submucosa, and proper muscle annotations were added under tissue layer as a child layers. The Colon Resection Project consisted of five separate projects: 1. Invasive tumor, 2. Tissue, and Tissue sublayers-1 3. Tissue sublayers 2 “Dark Tissue and Light Tissue” [Light Tissue represents the Subserosa layer of the colon, and Dark tissue represents all the other layers of the colon (mucosa, submucosa, and muscularis propria). Dark and Light Tissues were created to make a reliable distinction between subserosa and serosa and other layers to avoid false positives of subserosa in the submucosa layer because of the similarity of histomorphology], 4. Mucosal Subregions (Mucosa, Submucosa, Muscularis mucosa), 5. Vessels, and Lymph nodes. Supplementary Table S1 shows the trained WSI dataset for each Colon Resection Project. The same whole mount slide images were taught separately for all five separate projects. Ultimately, we made two projects. Only the Vessels and Lymph node Project was separated from the others. The algorithm consisted of three layers and four classes: 1-Tissue, 2-Mitosis, 3a-Atypical Mitosis, and 3b-Typical Mitosis for the Mitosis Project. Object detection was used for the mitosis, atypical mitosis, and typical mitosis layers. The mitosis layer was built to exclude mitosis mimics like apoptosis, necrotic tumor cells, stromal cells, etc. Annotations were made by a pathologist for each classifier in all three projects (Figure 2). Supplementary Table S2 shows the number of total semantic segmentation areas and objects that were annotated for each project and each layer.

Image analysis by an AI model was hierarchically conducted from the top layer to the final layer. In the case of our mitosis project, the first layer was designed as a binary semantic segmentation model that detects tissue regions. The second layer was an object detection model that counted mitosis within the tissue regions identified by the first layer. Finally, the detected mitotic cells were classified into typical and atypical by the third layer.

The accuracy of the trained AI model was calculated based on verification statistics, which were derived from the comparison of the AI-model predictions to the original input training annotations for each feature (false positive, false negative, total error, precision, sensitivity, and F1-Score). The F1 score is a statistical measure utilized to quantify the degree of overlap between two data sets [26] and represents the harmonic average of precision and sensitivity. If the area error was higher than 5%, and the confidence was less than 80%, the tested results were evaluated, then annotations were added or modified by a pathologist, and the AI model was retrained. These test-to-retrain processes were repeated until all layers reached satisfactory accuracy.

### Training, testing, and validation

In view of our future projects, all annotations were made by a pathologist, after AI models were created according to the corresponding pathological histological structures. The size and number of annotations were decided by the application specialist and pathologist together in each training round and proceeded accordingly. The cloud-based platform provided results in the “Verification and Validation” tool in comparison to the human-generated annotations, after the analysis. The results included false positives (regions that were annotated, and were found in the verification); percentage of the total area of all training regions-detection of non-invasive areas as invasive tumors in breast and colon tumors, false negatives (regions that were annotated and were not found in the verification), percentage of the total area of all training regions-undetected invasive areas, precision (space-dependent overlap of analysis results with human annotations), sensitivity (space-independent overlap of analysis results with annotations), and F measure (the harmonic mean of precision and sensitivity).

When the AI models achieved satisfactory accuracy, we released the latest models and tested them on untrained images. If the results were not sufficiently accurate, we did not release the model and kept training. Our criteria for success was to reach at least 90% of the F1 score. Once we reached satisfactory accuracy according to ground-truth pathologists the area error was less than 5% and the confidence was more than 80% for the AI models, with the validators agreeing on at least “very good” accuracy; therefore, we stopped training. All results were exported to be used as annotations for our applications.

Validators were invited to evaluate the results and rate the accuracy of the AI model for each project. Three pathologists were used for the evaluation of specific ROIs for each breast, mitosis, and colon project (Merve Basar, Irem Isgor, and Hulya Sahin Ozturk). They scored each feature blindly and independently of each other and the AI model. The validators evaluated the analysis results as a percentage with the classification, which we divided into four categories. The features of the segmentation area were rated for accuracy using the following scale: 1. Perfect or nearly perfect accuracy (95%–100%, no significant errors) 2. Very good accuracy (80%–95%, only minor errors) 3. Good accuracy (70%–80%, significant errors but still capturing the feature well) 4. Insufficient accuracy (less than 70%, significant errors compromising feature recognition).

## Results

### Breast project

**FIGURE 3:**
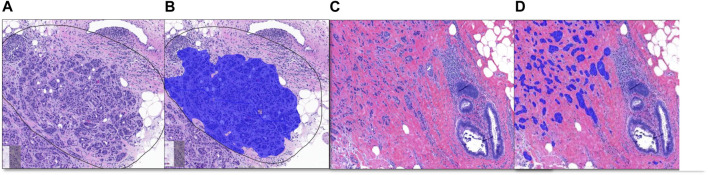
Breast Project results [From left to right **(A–D)**]. **(A, C)** are untrained images. **(A)** includes selected ROI (black line) for validation. **(B, D)** are image analysis results. **(B)**: Invasive tumor detection (blue) in selected ROI, **(D)**: The non-invasive region consisting of benign mammary glands and the invasive tumor detection (blue) are shown together.

After four runs, our final training results for the breast project were 0.10% false positive, and 0.16% false negative for the tissue layer, 0.56% false positive, and 0.73% false negative for invasive and non-invasive layers. Table 2 shows the results for each layer of the Breast Project, and Figure 3 shows the image analysis results for invasive carcinoma.

**TABLE 2:** Breast project verification and validation results.

	Precision (%)	Sensitivity (%)	F1 score (%)	Area error (%)
Tissue	99.60	99.34	99.47	0.26
Invasive	92.26	96.84	94.49	1.12
Non-invasive	99.65	98.73	99.19	1.45

#### External validator results of breast project

**FIGURE 4:**
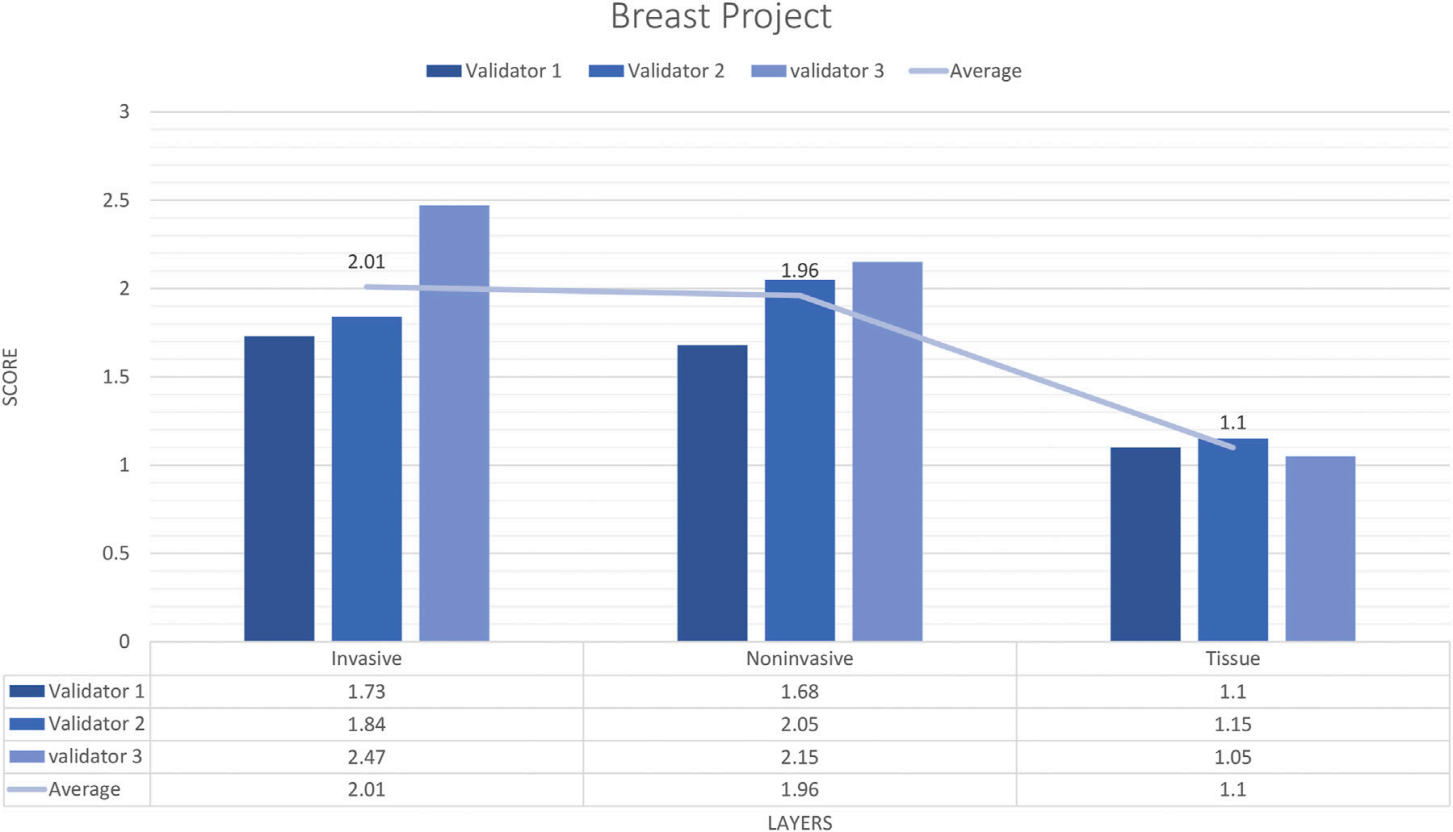
Breast carcinoma project external validator results. The evaluation of tissue, non-invasive, and invasive carcinoma detection in breast carcinoma cases by three pathologists. Segmentation area features were rated for accuracy using the following scale: 1. Perfect or nearly perfect accuracy (95%–100%, no significant errors) 2. Very good accuracy (80%–95%, only minor errors) 3. Good accuracy (70%–80%, significant errors but still captures the feature well) 4. Insufficient accuracy (less than 70%, significant errors compromising feature recognition). The average value for tissue and invasive carcinoma detection is in the range of 1 (Perfect or nearly perfect accuracy), and non-invasive layer detection is in the range of 2 (very good accuracy).

In total, 9 untrained images including 19 ROIs were sent to three pathologists. The external validator results showed that rating scores for invasive carcinoma (2) were in the range of “very good,” for tissue (1.1) and for non-invasive (1.9) were in the range of “very good” to “perfect or nearly perfect” (Figure 4). However, the median values were Score 1 (Perfect or nearly perfect accuracy) for Tissue and Invasive tumors and Score 2 (Very good accuracy) for Non-invasive layers.

### Colon ESD project

The final AI model results were 0.18% total area error, 0.12% false positivity, 0.06% false negativity, 99.36% precision, 99.71% sensitivity, and 99.54% F1 score for tissue class, 3.47% total area error, 0.36% false positivity, 0.51% false negativity, 96.74% precision, 95.42% sensitivity, 96.08%, F1 score, and 0.87% area error for tissue subregions. By looking a Table 3 we can see the other results for each layer.

**TABLE 3:** Colon ESD project tissue sublayers verification and validation results.

	Precision (%)	Sensitivity (%)	F1 score (%)	Area error (%)
Mucosa	96.80	97.47	97.13	1.40
Muscularis mucosa	84.65	84.63	84.64	0.56
Submucosa	98.06	92.98	95.46	1.41
Proper muscle	97.04	99.11	98.06	0.09

#### External validator results of the colon ESD project

**FIGURE 5:**
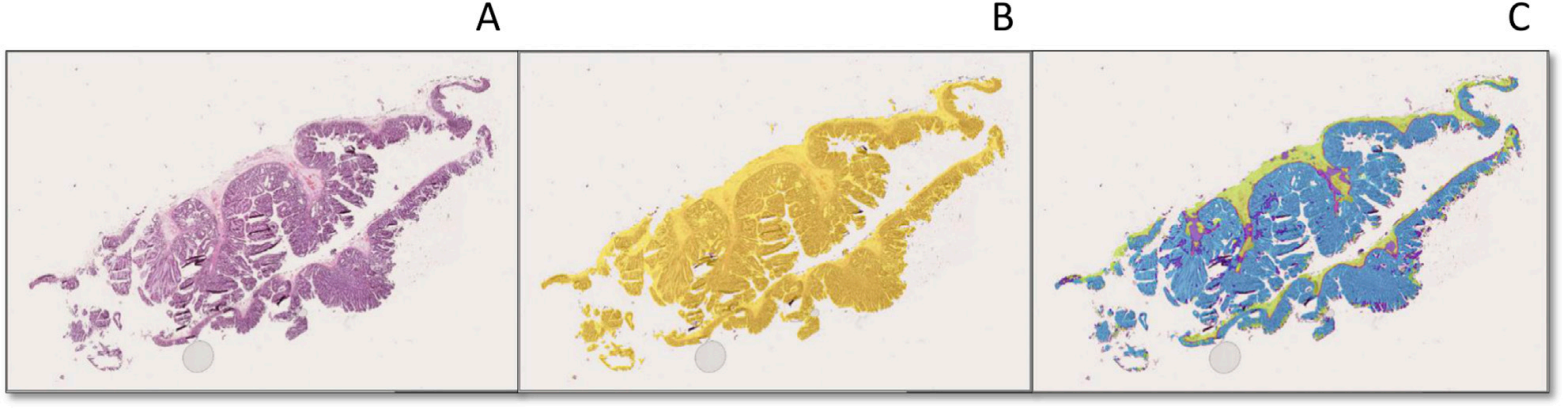
Tissue and subregion recognition feature of the ESD specimens by AI model **(A–C)**. **(A)**: An original H&E stain (×20 magnification) showing low-grade dysplasia in a colon ESD specimen; **(B)** and automated tissue (yellow) and **(C)** and automated colonic layer segmentation (mucosa: blue, muscularis mucosa: red, submucosa: green) original magnification [**(B, C)**, ×20].

This AI model was validated by three reviewers. Specifically, the reviewers were asked to draw annotations on the 30 validation regions. Their annotations were compared using intersection-over-union analysis against the AI model, on a pixel-by-pixel basis. Once the AI model performance reached 80% confidence, it was used to analyze the remaining 27 WSIs from the dataset. The final AI model showed a false positive rate of 0.18%, a false negative rate of 0.29%, a precision of 97.41%, a sensitivity of 95.89%, and an F1 score of 96.65%, respectively. External validation showed a false positive rate of 0.15%, a false negative rate of 0.058%, a precision of 92.01%, a sensitivity of 95.91%, and an F1 score of 97.57%, respectively. The image analysis generated by the final AI model showed correct discrimination of each layer with high confidence (Figure 5).

### Colon resection projects

**FIGURE 6:**
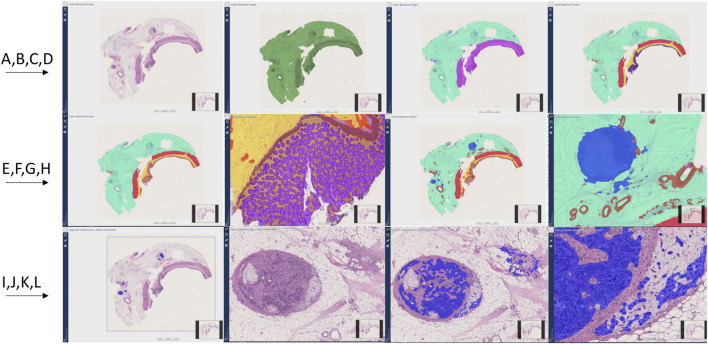
Colon resection project steps and image Analysis results **(A–J)**. First row from left to right **(A–D)**, Second row from left to right **(E–H)**, Third row from left to right **(I–L)**. **(A)**: Original whole slide image H&E stain (×20 magnification), **(B)**: Tissue detection (Green) **(C)**: Light and dark tissue detection [Light tissue (Subserosa and serosa): Light green, Dark tissue (muscular propria, submucosa, and mucosa): Purple] **(D)**: Subserosa and serosa (green), muscularis propria (red), submucosa (yellow), mucosa (purple) detection **(E, F)**: Mucosa subregions detection: Epithelium (Purple), Lamina propria (Yellow), muscularis mucosa (Brown) **(G, H)**: Vessel and lymph node detection (Red: Vessels, Blue: Lymph nodes**) (I)**: Invasive tumor detection (Blue) **(J)**: Metastatic lymph node and tumor focus in subserosa H&E, **(K, L)**: Invasive tumor detection (Blue) in metastatic lymph node and serosal fat tissue.

All layers showed over 90% F1-scores. Supplementary Table S3 shows the performance of the final AI model results for each layer and their child layers compared to the original annotations for the colon resection project. Figure 6 shows all detected layers by the AI models.

#### External validator results of the colon resection project_1

**FIGURE 7:**
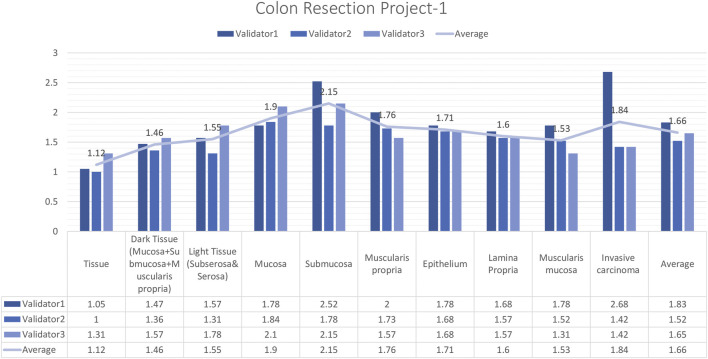
Colon Resection Project-1 External Validators Results. Scoring of all layers by three pathologists. Segmentation area features were rated for accuracy using the following scale: 1. Perfect or nearly perfect accuracy (95%–100%, no significant errors) 2. Very good accuracy (80%–95%, only minor errors) 3. Good accuracy (70%–80%, significant errors but still captures the feature well) 4. Insufficient accuracy (less than 70%, significant errors compromising feature recognition). The median value of vessel and lymph node detection is in the range of 1(Perfect or nearly perfect accuracy).

In total, 15 images and 19 ROIs were evaluated for Colon Resection Project_1 by three experienced pathologists. The results showed that the rating scores for tissue (1.12), dark tissue (1.47), light tissue (1.56), mucosa (1.91), muscularis propria (1.77), the epithelium (1.71), lamina propria (1.61), muscularis mucosa (1.54), and invasive carcinoma (1.84), were in the “perfect or nearly perfect” range and submucosa (2.15) was in the “very good” range (Figure 7). The most frequently assigned score in terms of average and median value was Score 1 (Perfect or nearly perfect) for all layers.

#### External validator results of the colon resection project_2

**FIGURE 8:**
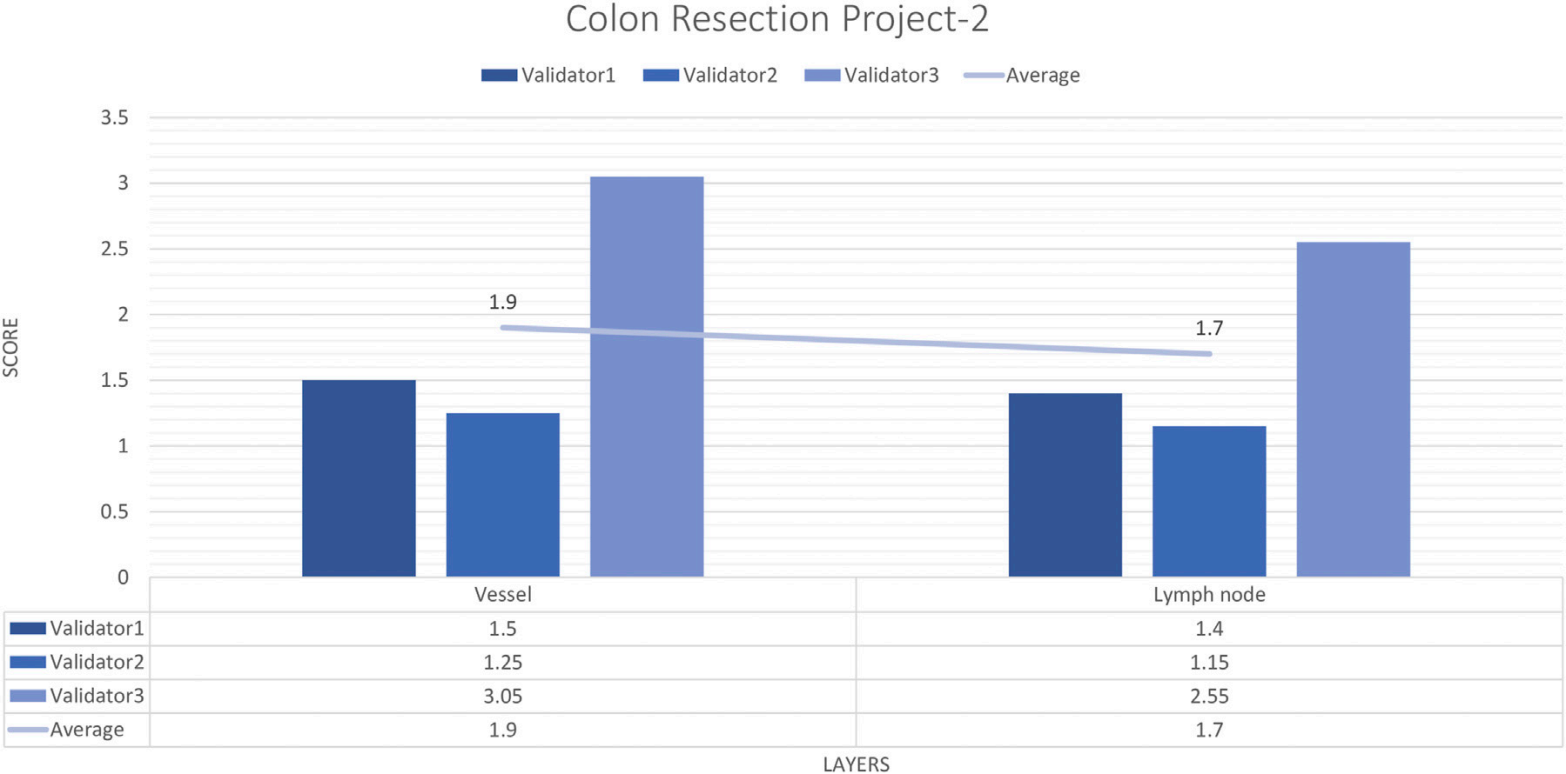
Colon Resection Project-2 external validators results. Evaluation of vessel and lymph node detection by three pathologists. Segmentation area features were rated for accuracy using the following scale: 1. Perfect or nearly perfect accuracy (95%–100%, no significant errors) 2. Very good accuracy (80%–95%, only minor errors) 3. Good accuracy (70%–80%, significant errors but still captures the feature well) 4. Insufficient accuracy (less than 70%, significant errors compromising feature recognition). The median value of vessel and lymph node detection is in the range of 1 (Perfect or nearly perfect accuracy).

For each vessel and lymph node project, 16 images and 20 ROIs were evaluated by three experienced pathologists. The external validator results showed that the rating scores for vessels (1.9) and lymph nodes (1.4) were in the “perfect or nearly perfect” range. The most frequently assigned score in terms of average and median value was Score 1 (Perfect or nearly perfect accuracy) (Figure 8).

### Mitosis project

**FIGURE 9:**
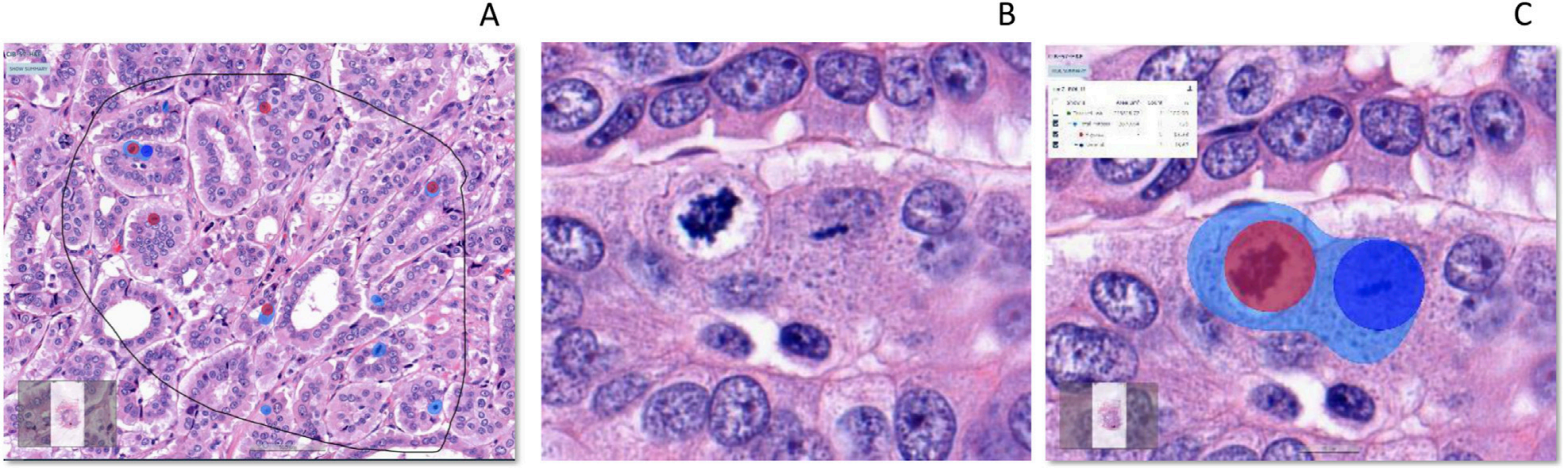
Detection of total mitosis, atypical and typical mitosis [From left to right **(A–C)**]. **(A)**: H&E, Invasive carcinoma breast, Selected ROI, **(B)**: Atypical and typical mitosis **(C)**: Detection of mitosis (Light Blue), Atypical mitosis (Red Circle) and Typical mitosis (Dark Blue Circle). IA, Image analysis; H&E, hematoxylin, and eosin.

Atypical and typical mitosis layers showed over 97% F1-scores. Table 4 shows the results of the mitosis project tree for each layer and class. The algorithm captured both typical and atypical mitotic figures (Figure 9).

**TABLE 4:** Mitosis project verification and validation results.

	Precision (%)	Sensitivity (%)	F1 score (%)	Area error (%)
Tissue	99.59	99.79	99.69	0.16
Total mitosis	67.85	97.97	80.18	0.17
Typical mitosis	97.74	97.07	97.40	
Atypical mitosis	98.18	97.19	97.68	

#### External validator results of the mitosis project

**FIGURE 10:**
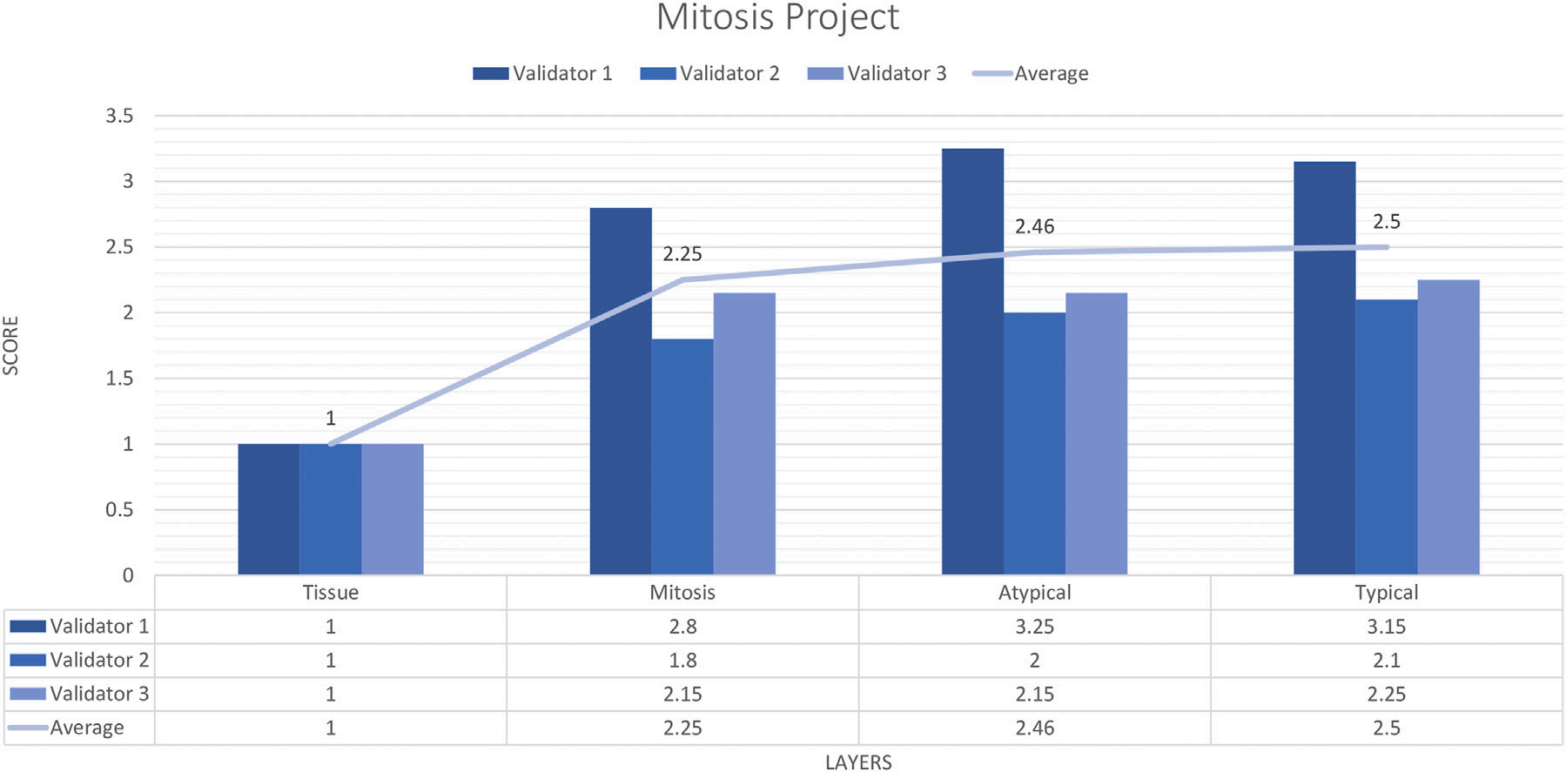
Mitosis project external validators results. Evaluation of tissue, mitosis, atypical and typical mitosis detection by three pathologists. Segmentation area features were rated for accuracy using the following scale: 1. Perfect or nearly perfect accuracy (95%–100%, no significant errors) 2. Very good accuracy (80%–95%, only minor errors) 3. Good accuracy (70%–80%, significant errors but still captures the feature well) 4. Insufficient accuracy (less than 70%, significant errors compromising feature recognition). The average value for tissue is in the range of 1 (Perfect or nearly perfect accuracy), and the detection of mitosis, atypical mitosis, and typical mitosis is in the range of 2 (very good accuracy).

Three pathologists evaluated 20 carefully selected ROIs in 4 WSIs. The external validator results showed that the rating scores for tissue (1) were in the “perfect or nearly perfect” range, but mitosis (2.83), atypical mitosis (2.45), and typical mitosis (2.5) were rated between “good and very good” accuracy. The highest score given by the three pathologists on average was 2 (Very good accuracy) for the Mitosis layer, Atypical Mitosis, and Typical Mitosis, and 1 (Perfect accuracy) for the Tissue layer. Therefore, the evaluation of all external validators as “total mitosis, atypical, and typical mitosis detection by AI” was classified in the “Very good” range. The validators agreed that AI could detect mitosis with at least 80%–95% accuracy (Figure 10).

## Discussion

AI models were built by pathologists with the help of application specialists, depending on the significant findings of histomorphology for each tissue. Models were trained and tested individually until the application reached reliability according to its own standards determined by pathologists, and then models include selected specific ROIs shared with validators (external pathologists) for validation. Finally, external pathologists who evaluated the models rated each model as having at least very good accuracy (80%–95%, only minor errors). Our findings showed that we were able to extract clinically relevant information from H&E slides with neural networks in line with the literature [2, 27]. AI algorithms exist in the literature that show that all tiles in each heatmap were assigned as hotspots for cancer detection [28] or any other histologic parameters [29]. Instead of using hot spots to recognize objects or segments, the algorithm we employed in our study displays the entire picture of histomorphology with extremely clear boundaries. It also examined each learned layer by separating it from other areas that the pathologists are not interested in. To use an example, children’s painting styles in coloring books vary according to their age. As they age, the frequency of coloring outside the lines in a coloring book rises. Through advanced analyses, our algorithm makes it possible to add molecular studies into these more accurate sharp boundaries in future projects, such as our breast project, in accordance with its morphology analyses.

In the Breast project, 59 images were trained with 18,777 iterations, the CNN complexity was set to extra complex, and the field of view was 100 µm. False positive areas consisted of desmoplastic tumor stroma, proliferative endothelial cells, and some empty spaces in tumor clusters. The location of false positive areas in the algorithm was the *in situ* area. In total, 54 untrained images and 10 benign breast tissues were tested by the final model of AI. In the benign 10 breast tissue images the false positive detection of invasive carcinoma ranged from 0.02% to 1.48%. Some groups of cells with hyperchromatic nuclei and areas of adenosis were detected as invasive carcinoma at an average rate of 1%. Considering that there are areas of cancer that can be detected by AI that may be overlooked by pathologists in some studies [30], it appears that this rate can be reduced to 0% in future studies. While two pathologists evaluated the diagnosis of invasive carcinoma by AI as nearly perfect (95%–100% accuracy) in all ROIs, the 3rd pathologist focused on Score 2 (80%–95% accuracy) in this evaluation. We checked and learned that the two validators who scored in a similar way were trained in the same medical school, whereas the third pathologist was educated in a different medical school. This is a good example of the need for artificial intelligence, which can provide an objective solution to the subjective perspective gained through education while making the diagnosis. Ultimately, three pathologists agreed that AI can detect breast-invasive tumors with at least 80%–95% accuracy.

Three different scanners were used for breast carcinoma, but we could not get satisfactory results due to low-quality images. We used image augmentation tools, but it was not enough. These images needed more annotations to detect invasive carcinoma areas compared to images from other scanners. Our results show that scanner and image quality affect the detection by AI, but we know it is fixable if we teach the AI when the resolution changes. Cross-verification of different scanners and different resolutions is beyond the scope of this publication. We need the AI to function for all images of our scanner lineup, but the AI algorithm is not required to support all scanners except our own in this study, as we used available scanners in real-world situations and it was sufficient to create accurate models and verify the results. For the mitosis detection model, we did not use 20× magnification (0.5 um/pixel resolution). We trained the AI until it could understand all formats. We see the potential for future evaluation between scanners and resolutions with the goal of AI becoming more objective and supporting all digital platforms to make a more accurate diagnosis.

Although the muscularis mucosa is a sublayer of the mucosa, it was used as a separate layer from the mucosa in the ESD Project due to its importance in terms of depth of invasion. However, when the results of the tissue and mucosa layers were examined after 53 images were trained, only the F1 score of the muscularis mucosa was less than 90% (84%). As for the colon resection project, the F1 scores of all colon project layers, including the muscularis mucosa, were above 90%. The differences were as follows: the ESD project was the only project where the evaluators made their own annotations (they were not pathologists), the colon resection project was created and trained by the pathologist for histologically correct positioning of the muscularis mucosa, and more importantly, the trained area was 36 times larger (Supplementary Table S2). A pathologist is vital to the success of the digital image analysis project, as the denotative analysis algorithm could not have been invented without their specialized expertise [31].

In the colon resection project tissue detection was as good as in the breast and ESD projects. The AI was able to detect any type of tissue layer, regardless of which organ it belonged to. In terms of accuracy, only the submucosal layer detection scoring was in the range between very good and good accuracy, while the other layers in the colon resection project were in the range of perfect accuracy. The vascular structures and loose connective tissue histology of the submucosa and subserosa are the same. Due to this similarity, we obtained false positive and false negative results in the separation of the two layers. To eliminate this overlap and confusion, we divided the colonic layers into two categories: light tissues as subserosa and dark tissues as other layers. Although we separated the subserosa from the submucosa, there were still negative areas and missing parts in the submucosal layer of the colon. However, it was satisfactory that we achieved high accuracy in the subserosa layer, which has an important place in prognosis and staging in the context of understanding tumor infiltration, detecting vascular invasion, and catching metastatic lymph nodes, and tumor deposits.

In total, 22 images were trained with 5,000 iterations, with CNN complexity set to extra complex and 50 µm field of view in the invasive carcinoma detection in the colon project. After 5 runs we reached the accurate analysis results. In the first model, false positive cells were located in normal mucosa. We changed the FOV (50 µm) and complexity (extra) by comparing the false positive areas between the AI models. With a lower FOV (200 µm) and complexity (very), fewer false positive cells were detected in normal mucosa. In the new model, false positives were found in the tumor stroma and lumen, and AI was able to separate the invasive tumor region from the non-invasive region.

The results of the lymph nodes and vessels project showed high accuracy in identifying vessels and lymph nodes. We used whole shape annotations to train for vessels and lymph nodes in the subserosa and serosa layers. The results showed that the detection was nearly perfect. The challenge for AI was that the muscularis propria layer also consisted of muscles like the vessel walls. In addition, we tried to do annotations with all the construction drawings for vessels. The handicap for lymph node identification was the difficulty in distinguishing epithelial cells in the mucosa. However, by using high iterations and adding background annotations, we fixed the majority of these problems in 4–5 training rounds, and the true positive rates became quite high.

Mitotic counting is a time-consuming process in pathology reporting, but it is just as important in nearly all carcinoma types to determine grade and prognosis. Even when counted again by the pathologist, there is a difference in mitotic rates, while it is an indisputable fact that this difference between pathologists is obvious [32]. We would like to implement clinical to count mitosis for pathologists to help to avoid time consuming processes. It is important to determine the best area for mitotic counting. A study showed that the use of High Power Fields (HPF) should be abandoned and replaced by standardized international (SI) units; the use of square millimeters (mm^2^) provides a properly standardized unit for area measurement, and the calculation of mitosis per mm^2^ becomes easier. The data obtained are not related to the microscope and magnification used [33]. The article cited referred to digital platforms that facilitate the development of mitotic counting algorithms that use the evaluation of the entire tumor, as in our algorithm, indicating that we can measure the mitotic index only in certain tumor types more accurately and find new ways to classify tumors. An AI application that will standardize the mitotic rate is very important for pathologists and other clinicians to determine treatment. In a recent study, a semi-automated image processing algorithm generated datasets directly from H&E and pHH3-stained tile images with a new U-Net-based mitosis detection model that learned mitotic features from annotated images proposed in a simple and highly efficient way according to classical methods without performing annotations [34]. The segmentation model was re-trained for the confusing cells called mitosis look-alikes (MLAs) to reduce false positives. In our study, the mitotic layer was the only layer with an F1 score below 90% in all projects. The mitotic layer was added later to the detection of atypical and typical mitotic objects to exclude artifacts. The aim was to reach an F1 score of 90% for atypical and typical mitosis. However, mitosis training was discontinued when accuracy in distinguishing atypical and typical mitosis increased. It should be recognized that it is not easy for AI to detect mitosis due to it being confused with dark hyperchromatic nuclei, lymphocytes, and other inflammatory cells, artifacts, pigments, and crushed cells [25]. However, the detection of atypical and typically distinctive mitosis offered an above-average detection rate in our study. One of our future plans is to define prognostic differences between atypical and typical mitotic counts and/or to determine whether there are any effects between these two classes in determining staging or grading in cancer types, specifically pancreatic cancer [35]. Another plan is to provide a measurement of mitotic count, rate, and index using mitosis detection by AI. Our shortcoming in this project, which serves as a step toward new research, was that we needed more data.

Additionally, as a part of future work, we exported the analysis results from the Aiforia platform to an image server in a JSON format. Because the exported files contain only coordinates of an outline of segmented or detected regions, each region was reconstructed on the local computer by our software, which confirmed that the segmentation results could be successfully reconstructed on the local computer. Based on this result, the analysis result obtained by the AI developed on the Aiforia platform may be used on other applications. For example, in the case of the breast cancer assessment, immunohistochemistry (IHC) and fluorescence and bright field *in situ* hybridization (ISH) slides are used together with H&E slides to determine the human epidermal growth factor receptor 2 (HER2) status. If invasive areas are automatically identified on H&E images by an AI model, an additional in-house machine-learning-based image analysis application may help in the subsequent assessment of IHC, and ISH images. This would reduce manual tedious operations and replace the assessment from subjective to objective. In addition, if AI accomplishes adequate accuracy on the Aiforia platform, these results may be used as annotations for additional development of in-house image analysis algorithms and/or AI models.

Some of our 3D pathology imaging applications use micro-computed tomography (Micro CT) to scan entire FFPE blocks or resected tissue non-destructively. Whole Block Imaging (WBI) and Whole Tissue Imaging (WTI) by a Micro-CT scanner are novel non-invasive imaging techniques that detect histomorphological features [36]. A recent study proposed 3D vessel detection using a deep neural network for micro-CT images. In addition to vessel detection, the purpose of the study was to detect tumor lesions, measure tumor invasion depth, and identify metastatic lymph nodes [37]. To improve the efficiency of this processing, it is necessary to integrate all the functions by applying methods such as ours, which were created for the Colon Project. We believe that by using AI-cloud-based platforms, we will be able to reach at least 80% accuracy for histomorphological features of samples, which will allow us to be able to integrate the 3D imaging tools and provide a broad spectrum, giving the pathologist the freedom to use all the tools in the process of achieving the most accurate diagnosis.

Currently, 3D imaging, and image analysis applications are highly desired. Some research groups have proposed machine-learning-based 3D analysis applications and the complexity of annotation has been pointed out as an important problem. In particular manual annotations of hundreds of serial sections of the same block is an extremely time-consuming and laborious task. If the developed AI model works on 3D imaging modalities such as micro-computed tomography or light sheet microscopy, we will develop an efficient AI model for 3D image analysis more easily. It should be kept in mind that specific cross- or multimodal image analysis strategies are required to seamlessly implement the deployment of 3D imaging devices and their integration with H&E images.

## Conclusion

This study aimed to develop a deep-learning segmentation algorithm that can define invasive carcinomas in the breast and colon, identify colonic layers in colon specimens, and distinguish between atypical and typical mitoses. The final step was to validate the output against interpretations by a pathologist and in an independent test cohort and finally to use the algorithm as an application in our software programs.

Our developed AI models showed excellent performance for tissue detection in all organ types. The AI models identified tumor areas well even in the presence of different tumor grades and *in situ* areas, in breast carcinomas, detected the invasive carcinoma and colonic layers, and recognized vessels and lymph nodes in colon specimens.

We will continue to create a new project for colorectal carcinoma resection specimens to teach AI to define prognostically significant findings in whole-mount images to apply it to Whole block 3D imaging and use the exported data from breast carcinomas to achieve the IHC/ISH assessment system.

## Data Availability

The original contributions presented in the study are included in the article/Supplementary Material, further inquiries can be directed to the corresponding author.
