# Supplementary material for: Artificial intelligence-based automated determination in breast and colon cancer and distinction between atypical and typical mitosis using a cloud-based platform
Source: Pathol Oncol Res. 2024 Oct 30;30:1611815. doi: 10.3389/pore.2024.1611815 (PMC11557341; doi:10.3389/pore.2024.1611815)
Supplement: Supplementary file 1 [file DataSheet1.docx]

**Supplementary Table 1.** Trained, tested, and validated Whole Mount Images (WMIs) in Colon Resection Project subgroups.

| **Colon Resection Project** | **Trained WMI** | **Tested WMI** | **Validated WMI** |
| --- | --- | --- | --- |
| **Tissue** | 17 | 6 | 15 (19 ROIs) |
| **Tissue subregions** | 23 | 21 | 15 (19 ROIs) |
| **Tissue subregions 2** | 33 | 10 | 15 (19 ROIs) |
| **Mucosa subregions** | 19 | 6 | 15 (19 ROIs) |
| **Invasive carcinoma** | 51 | 4 | 15 (19 ROIs) |
| **Vessel and lymph nodes** | 57 | 8 | 16 (20+20 ROIs) |
| **Total WMI** | 200 | 55 | 91 (135 ROIs) |

**Supplementary Table 2.** Annotated areas and objects for each project

|  | **Annotated Area (mm²)** | **Annotated Object** |
| --- | --- | --- |
| **Breast Project Total** | 415,003 |  |
| Tissue | 101,3 |  |
| Invasive Carcinoma | 10,802 |  |
| Non-Invasive | 91,251 |  |
| **Mitosis Project Total** |  | 2520 |
| Typical Mitosis |  | 1024 |
| Atypical Mitosis |  | 1496 |
| **Colon ESD Project Total** | 779,121 |  |
| Tissue | 126,549 |  |
| Mucosa | 0,901 |  |
| Submucosa | 1,393 |  |
| Muscularis mucosa | 0,058 |  |
| Proper Muscle | 0,929 |  |
| **Colon Resection Projects** |  |  |
| **Tissue&Tissue sublayers Total** | 1479,277 |  |
| Tissue | 749,128 |  |
| Mucosa | 44,519 |  |
| Submucosa | 95,183 |  |
| Muscularis Propria | 191,203 |  |
| Subserosa | 954,985 |  |
| **Tissue Subregions-2 Total** | 2909,037 |  |
| Light Tissue | 1590,835 |  |
| Dark Tissue | 1214,262 |  |
| **Mucosa subregions Total** | 282,738 |  |
| Epithelium | 4,424 |  |
| Lamina Propria | 2,254 |  |
| Muscularis mucosa | 2,143 |  |
| **Vessel&Lymph Nodes Total** | 662,24 |  |
| Vessel | 104,22 |  |
| Lymph Node | 130,432 |  |
| **Invasive Carcinoma Total** | 662,24 |  |
| Invasive Carcinoma | 12,336 |  |

**Supplementary Table 3.** Colon Resection Project Verification & Validation Results

| Colon resection project | Total area error (%) | False positive (%) | False negative (%) | Precision (%) | Sensitivity (%) | F1-score (%) | Area error (%) |
| --- | --- | --- | --- | --- | --- | --- | --- |
| **1-Tissue** | 0.14 | 0.10 | 0.04 | 99.80 | 99.92 | 99.86 | 0.14 |
| **2-Mucosa subregions** | 0.36 | 0.07 | 0.05 | 94.6 | 95.96 | 95.32 | 0.12 |
| Epithelium | 7.06 | 3.16 | 3.90 | 96.81 | 96.10 | 96.46 | 0.13 |
| Lamina propria | 12.43 | 6.88 | 5.55 | 93.21 | 94.45 | 93.83 | 0.14 |
| Muscularis mucosa | 10.70 | 8.28 | 2.42 | 92.18 | 97.58 | 94.80 | 0.09 |
| **3a-Tissue subregions** | 3.68 | 0.36 | 0.56 | 98.43 | 97.55 | 97.99 | 0.92 |
| Mucosa | 6.40 | 4.74 | 1.66 | 95.40 | 98.34 | 96.85 | 0.23 |
| Submucosa | 10.16 | 2.96 | 7.20 | 96.91 | 92.80 | 94.81 | 0.90 |
| Muscularis propria | 3.47 | 1.71 | 1.76 | 98.29 | 98.24 | 98.26 | 0.51 |
| Subserosa | 3.16 | 1.16 | 2.00 | 98.83 | 98.00 | 98.42 | 2.04 |
| **3b- Tissue subregions-2** | 1.03 | 0.07 | 0.44 | 99.85 | 99.09 | 99.47 | 0.52 |
| Dark tissue | 0.81 | 0.21 | 0.61 | 99.79 | 99.39 | 99.59 | 0.24 |
| Light tissue (subserosa &serosa) | 1.17 | 0.12 | 1.05 | 99.88 | 98.95 | 99.41 | 0.79 |
| **4-Invasive carcinoma** | 0.29 | 0.25 | 0.04 | 86.14 | 97.77 | 91.59 | 0.29 |
| **5-Vessel&**  **Lymph nodes** | 0.79 | 0.27 | 0.13 | 97.35 | 98.69 | 98.02 | 0.40 |
| Vessels | 7.38 | 5.04 | 2.34 | 95.10 | 97.66 | 96.36 | 0.65 |
| Lymph nodes | 1.29 | 0.80 | 0.49 | 99.20 | 99.51 | 99.36 | 0.14 |
